# Monosomy 18p

**DOI:** 10.1186/1750-1172-3-4

**Published:** 2008-02-19

**Authors:** Catherine Turleau

**Affiliations:** 1 Cytogénétique AP-HP et Inserm U781, Université Paris Descartes, Hôpital Necker-Enfants Malades, 75015 Paris, France

## Abstract

Monosomy 18p refers to a chromosomal disorder resulting from the deletion of all or part of the short arm of chromosome 18. The incidence is estimated to be about 1:50,000 live-born infants. In the commonest form of the disorder, the dysmorphic syndrome is very moderate and non-specific. The main clinical features are short stature, round face with short philtrum, palpebral ptosis and large ears with detached pinnae. Intellectual deficiency is mild to moderate. A small subset of patients, about 10–15 percent of cases, present with severe brain/facial malformations evocative of holoprosencephaly spectrum disorders. In two-thirds of the cases, the 18p- syndrome is due to a mere terminal deletion occurring *de novo*, in one-third the following are possible: a *de novo *translocation with loss of 18p, malsegregation of a parental translocation or inversion, or a ring chr18. Parental transmission of the 18p- syndrome has been reported. Cytogenetic analysis is necessary to make a definite diagnosis. Recurrence risk for siblings is low in *de novo *deletions and translocations, but is significant if a parental rearrangement is present. Deletion 18p can be detected prenatally by amniocentesis or chorionic villus sampling and cytogenetic testing. Differential diagnosis may include a wide number of syndromes with short stature and mild intellectual deficiency. In young children, deletion 18p syndrome may be vaguely evocative of either Turner syndrome or trisomy 21. No specific treatment exists but speech therapy and early educational programs may help to improve the performances of the children. Except for the patients with severe brain malformations, the life expectancy does not seem significantly reduced.

## Disease name/synonyms

Monosomy 18p, deletion 18p syndrome, 18p- syndrome, del(18p) syndrome, partial monosomy 18p, de Grouchy syndrome 1

## Definition/diagnostic criteria

Monosomy 18p refers to a chromosomal disorder resulting from the absence of all or part of the short arm of chromosome 18. It was reported in 1963 by the French geneticist Jean de Grouchy [1,2] and was the first example of a partial monosomy compatible with life. Clinical features typically include mild to moderate mental retardation, short stature, round face with short protruding philtrum, palpebral ptosis and large ears with detached pinnae. Cytogenetic analysis is necessary to make a definite diagnosis.

## Epidemiology

More than 150 patients have been reported worldwide and most cases are no longer subject to publication. The incidence of the disorder could be estimated as about 1:50,000 live-born infants. The female to male ratio is 3/2.

## Clinical description

Manifestations of the syndrome vary greatly from one patient to another [3,4] (Table 1). Pregnancy and delivery were mostly normal. Mean birth weight is in the low normal range.

**Table 1:** Main clinical features observed in Monosomy 18p syndrome

**Very frequent**
mental retardation (variable severity)
speech delay
short stature
**Frequent**
variable features of the holoprosencephaly (HPE) spectrum
ptosis
flat nasal bridge
wide mouth with short upper lip
small mandible
excessive caries
large, protruding ears
short, webbed neck
broad trunk
pectus excavatum
kyphoscoliosis

**Rare**
behavioral disorders
autoimmune diseases
alopecia
dystonia

### Physical appearance

**Figure 1:**
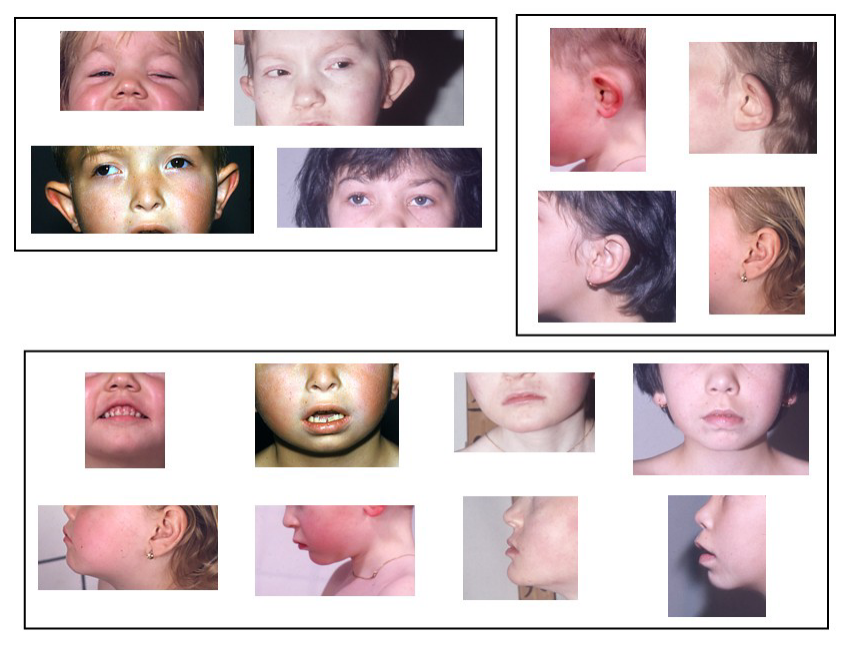
**Facial features in patients with Monosomy 18p syndrome.** Flat midface, mild ptosis, large ears with detached pinnae and short protruding upper lip are frequent findings.

Except for a small subset of patients who present with severe malformations pertaining to the holoprosencephaly spectrum, the diagnosis may not be evident at birth. It becomes easier at some years of age. Although the phenotype is not as characteristic as for other chromosomal syndromes, many patients have a certain resemblance (Figure 1). Typical patients are small, with a short neck and a characteristic posture: they stand with widespread legs and leaning slightly forward. Mild microcephaly may be present. The face is round, flat and expressionless, the nasal bridge is flat and broad, the palpebral fissures are horizontal, epicanthal folds, strabismus, and mostly ptosis of the eyelids when present are important features. Ptosis could be uni- or bilateral and may require surgical correction. The mouth is wide, the philtrum is rather short and protruding, Cupid's bow is blunted with a flat upper lip and the lower lip is often everted. The irregularly set teeth are of poor quality with significant caries, lateral incisors are sometimes missing, palate may be high arched. The chin is small and slightly receding in children, becoming normal or even protruding in adults. The ears are large, floppy, with detached pinnae and a hypoplastic anthelix, and are often low-set and posteriorly rotated. The hands are wide and short with phalanges of diminishing width, dermatoglyphics are not characteristic. A short, sometimes webbed neck, a low posterior hair line and a broad chest with widely spaced nipples or pectus excavatum are frequent signs which can be evocative of Turner syndrome.

Muscular hypotonia is very frequent. Puberty is normal in most cases and fertility is possible.

### Psychomotor delay

Mental retardation is usual. The intelligence quotient (IQ) varies between 25 and 75, being around 50 in most cases, although some patients have been reported with normal or borderline mental development. Speech delay is frequently present, and verbal and manual abilities are often highly dissociated. Marked slowness in motion and action has been noted [5,6]. Behavioral phenotypes such as autism or schizophrenia sometimes complicate the mental deficiency. Convulsive fits or electroencephalographic (EEG) disturbances are rarely observed [7].

### Holoprosencephaly-type defects

The main malformation is holoprosencephaly (HPE) which involves the abnormal development of the forebrain and the midface, and is associated with a large phenotypic spectrum [8]. Severe malformations of the brain associated with facial features such as cyplopia, cebocephaly, premaxillary agenesis, bilateral cleft lip and palate are present in 10–15 percent of cases with deletion 18p syndrome [9-11]. Milder forms include absent olfactory tracts and bulbs, agenesis of corpus callosum, hypopituitarism and minor facial features (hypo- or hypertelorism, flat nasal bridge) with/without brain malformation. A single central maxillary incisor as an abortive form of holoprosencephaly has been repeatedly observed in 18p- syndrome [12].

### Other malformations

Various skeletal deformities such as scoliosis and/or kyphosis, coxa vara, dislocation of the hip and feet deformities have been reported. In males, genital hypoplasia with small penis and cryptorchidism is occasionally observed. Cardiac malformations appeared to be relatively uncommon, observed in about 10 percent of patients, with situs abnormalities in some cases [13]. Various other malformations have been rarely or occasionally reported, often for deletion 18p secondary to an unbalanced translocation with a concomitant partial trisomy.

### Other abnormalities

In those patients with short stature, growth hormone (GH) deficiency is frequently found and may justify GH treatment [14]. Absence or reduction of serum immunoglobulin A (IgA) may be present [15]. Thyroiditis leading to insufficiency or thyrotoxicosis [16], juvenile diabetes and other auto-immune disorders have been reported.

Hypotrichosis simplex, total baldness or alopecia areata have been observed [9,17], as well as other rare cutaneous disorders such as keratosis pilaris and ulerythema ophryogenes [18,19]. Dystonia, a movement disorder, may appear in young adulthood [20,21].

### Genotype-phenotype correlations

Correlation between the breakpoints and the mental development of seven subjects suggests a critical region between p11.1 and p11.21, since three patients with a deletion distal to this point have normal or borderline mental development [6]. Mapping phenotypical traits have been also attempted from a small number of patients. Round face was tentatively mapped to the distal 1.6 Mb of chr 18 short arm, post-natal growth retardation and seizures to the distal 8 Mb, and ptosis and short neck to the proximal half of 18p [22]. Further studies are needed to confirm these results [23].

## Aetiology

Deletion 18p syndrome is due to the absence of all or part of the short arm of one chromosome 18. Parental karyotypes must be studied to determine if either is a balanced translocation carrier or has the unbalanced 18p- deletion.

Most cases (about 2/3) are *de novo *deletions. The short arm of chromosome 18 is about 16 Mb in size [24]. It is divided in three subbands: p11.1 adjacent to the centromere, p11.2 subdivided in p11.21, p11.22 and p11.23, and p11.3 subdivided in p11.31 and p11.32 [25]. A preferential breakpoint cluster at 18p11.1 has been suggested after study of 25 non-mosaic patients with *de novo *deletion of 18p and an apparent breakpoint cluster in the pericentromeric region on 18p with only 7/25 subjects with breakpoint outside [26]. In this study, maternal and paternal origin seemed to be equally common. No example of interstitial deletion has been reported to date.

Among other reported cases, many result from an unbalanced whole arm translocation occuring usually between the long arm of an acrocentric chromosome and the long arm of chr 18 and resulting in a karyotype with 45 chromosomes [27]. Other deletions 18p are the consequence of malsegregation of a balanced parental translocation with a variable breakpoint on 18p and are accompanied by a partial trisomy for another chromosome. Some cryptic subtelomeric deletions or translocations have been evidenced using subtelomeric screening [28,29].

Mosaicism or association with another aneuploidy are sometimes observed.

Familial transmission of 18p- from one of the parent to the child has been reported in at least six cases, most of them with a maternal transmission [9,30,31]. Deletion 18p may be in a homogeneous or a mosaic state in the parent, and intrafamilial clinical variability may be present.

Deletion of 18p appears sometimes as part of a ring 18 chromosome [32] or after recombination in a pericentric inversion leading to a 18p monosomy associated to a 18q trisomy [33].

### Relevant genes and loci

A critical region for holoprosencephaly, HPE4, has been defined on a molecular level to the most distal segment of 18p [11]. Mutations in the *TGIF *gene located on 18p11.3 have been shown to cause holoprosencephaly [34]. Hemizygosity of HPE4 does not automatically confer the phenotype of HPE, since only 10–15 percent of patients have features consistent with HPE, confirming that multiple genetic and environmental factors intervene in HPE spectrum phenotypes. This low concordance is much lower than those seen with other HPE loci.

An autosomal form of hereditary hypotrichosis simplex and a susceptibility locus for alopecia areata were identified on 18p [35,36]. DYT7, one of the loci for dystonia is known to be located on 18p [37].

Linkage studies have implicated the 18p11.2 region in susceptibility to bipolar disorders and schizophrenia with a parent-of-origin effect. The *GNAL *gene is an attractive candidate gene [38,39].

For all these disorders, the deletion 18p could unmask a recessive defect in the undeleted homologous chromosome or could lead to a loss of function of an autosomal dominant gene with low penetrance and/or variable expressivity.

## Diagnostic methods

It is not possible to base the diagnosis of deletion 18p syndrome merely on the phenotype and cytogenetic analysis is necessary to make a definite diagnosis. Diagnosis is usually done by karyotype analysis from peripheral blood. It is also possible in prenatal period from amniocytes or trophoblast cells. Systematic subtelomeric screening in mentally retarded patients had revealed only a low frequency of cryptic deletions or translocations involving 18pter [40]. However, specific subtelomeric FISH may be useful to confirm the diagnosis and may help to characterise partial deletions or cryptic translocations.

## Differential diagnosis

Differential diagnosis may include a wide number of syndromes presenting with short stature and mild mental retardation. In young children, deletion 18p may be vaguely evocative of either Turner syndrome or trisomy 21. In all cases, cytogenetic analysis allows the right diagnosis.

## Genetic counselling

For those cases that arise *de novo*, the recurrence risk for siblings is not significantly increased above that of the general population. However, prenatal diagnosis may be counselled because cryptic mosaicism may be present in one of the parent.

The recurrence risk is significant if a structural rearrangement is present in one of the parent. The most frequently observed parental rearrangement is balanced translocation, followed by pericentric inversion. In those cases, recurrence risk depends on the type of rearrangement in which chromosomes are involved and on the size of the rearranged segments. For some rearrangements, there is a high risk of either a monosomy or a trisomy for 18p.

If one of the parent is carrier of a 18p deletion, the risk of recurrence for siblings may be as high as 50 percent if the 18p deletion is present in a homogeneous state in the parent, or lower if the 18p deletion is present in a mosaic state.

## Antenatal diagnosis

Deletion 18p can be detected prenatally by amniocentesis or chorionic villus sampling and cytogenetic testing including FISH. This could be done when a parent is heterozygous for a balanced rearrangement involving 18p or carrier of a 18p deletion, following detection of a holoprosencephaly-type defect at sonography, or after the birth of a first affected child. Deletion 18p is rarely observed in first-trimester abortions suggesting that this imbalance is not selected against.

## Management including treatment

As for other chromosomal disorders, no specific treatment exists for deletion 18p syndrome, but early rehabilitative and educational interventions are recommended, mainly speech therapy, since the majority of patients have major speech problems and difficulties with speech articulation. Physical therapy for hypotonia should be advised.

## Prognosis

The prognosis is poor for those patients with severe brain malformations; most often they die in the newborn period. Survival does not seem to be reduced in patients with the commonest form of deletion 18p syndrome, in absence of severe malformations [41]. Developmental delay is the main concern. As some children may have average abilities in selected area, comprehensive developmental assessments and remedial special education programming should be proposed before a definite prognosis is determined [42].

## References

[B1] de Grouchy J, Lamy M, Thieffry S, Arthuis M, Salmon CH (1963). Dysmorphie complexe avec oligophrenie: deletion des bras courts d'un chromosome 17–18. C R Acad Sci.

[B2] de Grouchy J (1969). The 18p, 18q and 18 syndromes. Birth defects Orig Art Ser.

[B3] de Grouchy J, Turleau C (1984). Clinical Atlas of Human Chromosomes.

[B4] Schinzel A (2001). Catalogue of Unbalanced Chromosome Aberrations in Humans.

[B5] Faust J, Habedank M, Nieuwenhuijsen C (1976). The 18 p- syndrome. Report of four cases. Eur J Pediatr.

[B6] Wester U, Bondeson ML, Edeby C, Anneren G (2006). Clinical and molecular characterization of individuals with 18p deletion: a genotype-phenotype correlation. Am J Med Genet A.

[B7] Grosso S, Pucci L, Di Bartolo RM, Gobbi G, Bartalini G, Anichini C, Scarinci R, Balestri M, Farnetani MA, Cioni M, Morgese G, Balestri P (2005). Chromosome 18 aberrations and epilepsy: a review. Am J Med Genet A.

[B8] Cohen MM (2006). Holoprosencephaly: clinical, anatomic, and molecular dimensions. Birth Defects Res A Clin Mol Teratol.

[B9] Uchida IA, McRae KN, Ray M (1965). Familial short arm deficiency of chromosome 18 concomitant with arhinencephaly and alopecia congenita. Am J Hum Genet.

[B10] Münke M, Page DC, Brown LG, Armson BA, Zackai EH, Mennuti MT, Emanuel BS (1988). Molecular detection of a Yp/18 translocation in a 45, X holoprosencephalic male. Hum Genet.

[B11] Overhauser J, Mitchell HF, Zackai EH, Tick DB, Rojas K, Muenke M (1995). Physical mapping of the holoprosencephaly critical region in 18p11.3. Am J Hum Genet.

[B12] Taine L, Goizet C, Wen ZQ, Chateil JF, Battin J, Saura R, Lacombe D (1997). 18p monosomy with midline defects and a de novo satellite identified by FISH. Ann Genet.

[B13] Digilio MC, Marino B, Giannotti A, Di Donato R, Dallapiccola B (2000). Heterotaxy with left atrial isomerism in a patient with deletion 18p. Am J Med Genet.

[B14] Schober E, Scheibenreiter S, Frisch H (1995). 18p monosomy with GH-deficiency and empty sella: good response to GH-treatment. Clin Genet.

[B15] Leisti J, Leisti S, Perheentupa J, Savilahti E, Aula P (1973). Absence of IgA and growth hormone deficiency associated with short arm deletion of chromosome 18. Arch Dis Child.

[B16] Dharmaraj P, Grueters A (2006). The management of thyrotoxicosis in a pre-pubertal child with 18p deletion syndrome. Eur J Endocrinol.

[B17] Kantaputra PN, Limwongse C, Tochareontanaphol C, Mutirangura A, Mevatee U, Praphanphoj V (2006). Contiguous gene syndrome of holoprosencephaly and hypotrichosis simplex: association with an 18p11.3 deletion. Am J Med Genet A.

[B18] Nazarenko SA, Ostroverkhova NV, Vasiljeva EO, Nazarenko LP, Puzyrev VP, Malet P, Nemtseva TA (1999). Keratosis pilaris and ulerythema ophryogenes associated with an 18p deletion caused by a Y/18 translocation. Am J Med Genet.

[B19] Zouboulis CC, Stratakis CA, Gollnick HP, Orfanos CE (2001). Keratosis pilaris/ulerythema ophryogenes and 18p deletion: is it possible that the LAMA1 gene is involved?. J Med Genet.

[B20] Klein C, Page CE, LeWitt P, Gordon MF, de Leon D, Awaad Y, Breakefield XO, Brin MF, Ozelius LJ (1999). Genetic analysis of three patients with an 18p- syndrome and dystonia. Neurology.

[B21] Nasir J, Frima N, Pickard B, Malloy MP, Zhan L, Grunewald R (2006). Unbalanced whole arm translocation resulting in loss of 18p in dystonia. Mov Disord.

[B22] Brenk CH, Prott EC, Trost D, Hoischen A, Walldorf C, Radlwimmer B, Wieczorek D, Propping P, Gillessen-Kaesbach G, Weber RG, Engels H (2007). Towards mapping phenotypical traits in 18p- syndrome by array-based comparative genomic hybridisation and fluorescent in situ hybridisation. Eur J Hum Genet.

[B23] Portnoï MF, Gruchy N, Marlin S, Finkel L, Denoyelle F, Dubourg C, Odent S, Siffroi JP, Le Bouc Y, Houang M (2007). Midline defects in deletion 18p syndrome: clinical and molecular characterization of three patients. Clin Dysmorphol.

[B24] Nusbaum C, Zody MC, Borowsky ML, Kamal M, Kodira CD, Taylor TD, Whittaker CA, Chang JL, Cuomo CA, Dewar K, FitzGerald MG, Yang X, Abouelleil A, Allen NR, Anderson S, Bloom T, Bugalter B, Butler J, Cook A, DeCaprio D, Engels R, Garber M, Gnirke A, Hafez N, Hall JL, Norman CH, Itoh T, Jaffe DB, Kuroki Y, Lehoczky J, Lui A, Macdonald P, Mauceli E, Mikkelsen TS, Naylor JW, Nicol R, Nguyen C, Noguchi H, O'Leary SB, O'Neill K, Piqani B, Smith CL, Talamas JA, Topham K, Totoki Y, Toyoda A, Wain HM, Young SK, Zeng Q, Zimmer AR, Fujiyama A, Hattori M, Birren BW, Sakaki Y, Lander ES (2005). DNA sequence and analysis of human chromosome 18. Nature.

[B25] Mitelman F, ISCN (2005) (2005). An International System for Human Cytogenetic Nomenclature.

[B26] Schaub RL, Reveles XT, Baillargeon J, Leach RJ, Cody JD (2002). Molecular characterization of 18p deletions: evidence for a breakpoint cluster. Genet Med.

[B27] Wang JC, Nemana L, Kou SY, Habibian R, Hajianpour MJ (1997). Molecular cytogenetic characterization of 18;21 whole arm translocation associated with monosomy 18p. Am J Med Genet.

[B28] Horsley SW, Knight SJ, Nixon J, Huson S, Fitchett M, Boone RA, Hilton-Jones D, Flint J, Kearney L (1998). Del(18p) shown to be a cryptic translocation using a multiprobe FISH assay for subtelomeric chromosome rearrangements. J Med Genet.

[B29] Babovic-Vuksanovic D, Jenkins SC, Ensenauer R, Newman DC, Jalal SM (2004). Subtelomeric deletion of 18p in an adult with paranoid schizophrenia and mental retardation. Am J Med Genet A.

[B30] Rigola MA, Plaja A, Mediano C, Miro R, Egozcue J, Fuster C (2001). Characterization of a heritable partial monosomy 18p by molecular and cytogenetic analysis. Am J Med Genet.

[B31] Maranda B, Lemieux N, Lemyre E (2006). Familial deletion 18p syndrome: case report. BMC Med Genet.

[B32] Stankiewicz P, Brozek I, Helias-Rodzewicz Z, Wierzba J, Pilch J, Bocian E, Balcerska A, Wozniak A, Kardas I, Wirth J, Mazurczak T, Limon J (2001). Clinical and molecular-cytogenetic studies in seven patients with ring chromosome 18. Am J Med Genet.

[B33] Leonard NJ, Tomkins DJ, Demianczuk N (2000). Prenatal diagnosis of holoprosencephaly (HPE) in a fetus with a recombinant(18)dup(18q)inv(18)(p11.31q11.2)mat. Prenat Diagn.

[B34] Gripp KW, Wotton D, Edwards MC, Roessler E, Ades L, Meinecke P, Richieri-Costa A, Zackai EH, Massague J, Muenke M, Elledge SJ (2000). Mutations in TGIF cause holoprosencephaly and link NODAL signalling to human neural axis determination. Nat Genet.

[B35] Baumer A, Belli S, Trueb RM, Schinzel A (2000). An autosomal dominant form of hereditary hypotrichosis simplex maps to 18p11.32-p11.23 in an Italian family. Eur J Hum Genet.

[B36] Martinez-Mir A, Zlotogorski A, Gordon D, Petukhova L, Mo J, Gilliam TC, Londono D, Haynes C, Ott J, Hordinsky M, Nanova K, Norris D, Price V, Duvic M, Christiano AM (2007). Genomewide scan for linkage reveals evidence of several susceptibility loci for alopecia areata. Am J Hum Genet.

[B37] Klein C, Page CE, LeWitt P, Gordon MF, de Leon D, Awaad Y, Breakefield XO, Brin MF, Ozelius LJ (1999). Genetic analysis of three patients with an 18p- syndrome and dystonia. Neurology.

[B38] Vuoristo JT, Berrettini WH, Overhauser J, Prockop DJ, Ferraro TN, Ala-Kokko L (2000). Sequence and genomic organization of the human G-protein Golfalpha gene (GNAL) on chromosome 18p11, a susceptibility region for bipolar disorder and schizophrenia. Mol Psychiatry.

[B39] Corradi JP, Ravyn V, Robbins AK, Hagan KW, Peters MF, Bostwick R, Buono RJ, Berrettini WH, Furlong ST (2005). Alternative transcripts and evidence of imprinting of GNAL on 18p11.2. Mol Psychiatry.

[B40] Ravnan JB, Tepperberg JH, Papenhausen P, Lamb AN, Hedrick J, Eash D, Ledbetter DH, Martin CL (2006). Subtelomere FISH analysis of 11 688 cases: an evaluation of the frequency and pattern of subtelomere rearrangements in individuals with developmental disabilities. J Med Genet.

[B41] de Ravel TJ, Thiry P, Fryns JP (2005). Follow-up of adult males with chromosome 18p deletion. Eur J Med Genet.

[B42] Thompson RW, Peters JE, Smith SD (1986). Intellectual, behavioral, and linguistic characteristics of three children with 18p- syndrome. J Dev Behav Pediatr.

